# Identification, evolution, and expression partitioning of miRNAs in allopolyploid *Brassica napus*

**DOI:** 10.1093/jxb/erv420

**Published:** 2015-09-10

**Authors:** Enhui Shen, Jun Zou, Falk Hubertus Behrens, Li Chen, Chuyu Ye, Shutao Dai, Ruiyan Li, Meng Ni, Xiaoxue Jiang, Jie Qiu, Yang Liu, Weidi Wang, Qian-Hao Zhu, Boulos Chalhoub, Ian Bancroft, Jinling Meng, Daguang Cai, Longjiang Fan

**Affiliations:** ^1^Institute of Crop Sciences & Institute of Bioinformatics, College of Agriculture and Biotechnology, Zhejiang University, Hangzhou 310058, PR China; ^2^National Key Laboratory of Crop Genetic Improvement, Huazhong Agricultural University, Wuhan 430070, PR China; ^3^Department of Molecular Phytopathology and Biotechnology, Christian-Albrechts University of Kiel, Hermann Rodewald Str. 9, D-24118 Kiel, Germany; ^4^CSIRO Agriculture Flagship, Canberra, ACT2601, Australia; ^5^Organization and Evolution of Plant Genomes, Unité de Recherche en Génomique Végétale, Unité Mixte de Recherche 1165 (Institut National de Recherche Agronomique, Centre National de la Recherche Scientifique, Université Evry Val d’Essonne), Evry 91057, France; ^6^Centre for Novel Agricultural Products (CNAP), Department of Biology, University of York, Wentworth Way, Heslington, York YO10 5DD, UK

**Keywords:** allopolyploid evolution, *Brassica napus*, expression partitioning, microRNA.

## Abstract

The recently published genome of *Brassica napus* offers for the first time the opportunity to gain insights into the genomic organization and the evolution of miRNAs in oilseed rape. In this study, 12 small RNA libraries from two *B. napus* cultivars (Tapidor and Ningyou7) and their four double-haploid lines were sequenced, employing the newly sequenced *B. napus* genome, together with genomes of its progenitors *Brassica rapa* and *Brassica oleracea*. A total of 645 miRNAs including 280 conserved and 365 novel miRNAs were identified. Comparative analysis revealed a high level of genomic conservation of *MIRNA*s (75.9%) between the subgenomes of *B. napus* and its two progenitors’ genomes, and *MIRNA* lost/gain events (133) occurred in *B. napus* after its speciation. Furthermore, significant partitioning of miRNA expressions between the two subgenomes in *B. napus* was detected. The data of degradome sequencing, miRNA-mediated cleavage, and expression analyses support specific interactions between miRNAs and their targets in the modulation of diverse physiological processes in roots and leaves, as well as in biosynthesis of, for example, glucosinolates and lipids in oilseed rape. These data provide a first genome-wide view on the origin, evolution, and genomic organization of *B. napus MIRNA*s.

## Introduction

Small RNAs include two main types of small non-coding RNAs, microRNAs (miRNAs) and small interfering RNAs (siRNAs). miRNAs are endogenous 20–24 nt RNAs that can regulate gene expression via complementary binding to specific mRNAs and achieve their functions via post-transcriptional gene silencing in animals and plants (Voinnet, 2009; Rogers and Chen, 2013). The *MIRNA* gene is first transcribed into primary miRNA (pri-miRNA) by RNA polymerase II (Pol II) and then catalysed by endoribonuclease Dicer-like 1 (DCL1) in association with HYPONASTICLEAVES 1 (HYL1) and SERRATE (SE) proteins leading to the formation of a hairpin structure, or precursor miRNA (pre-miRNA). The pre-miRNA hairpin structure is further processed into a miRNA/miRNA* duplex, of which the mature miRNA is loaded onto the Argonaute (AGO) protein complex to execute its function (Vazquez *et al.*, 2010). miRNAs are involved in regulating many plant physiological processes including plant development and biotic and abiotic stress responses (Chen, 2009; Khraiwesh *et al.*, 2012; Sunkar *et al.*, 2012; Shen *et al.*, 2014; Zhang 2015).

*Brassica napus*, known as oilseed rape, is second only to soybean as an oil crop with a world production of over 60 million t (Bancroft *et al.*, 2011). *B. napus* is an allopolyploid (A_n_A_n_C_n_C_n_) species that evolved from the spontaneous hybridization of *Brassica rapa* (A_r_A_r_) and *Brassica oleracea* (C_o_C_o_) about 7500–12 500 years ago (Nagaharu, 1935; Chalhoub *et al.*, 2014). The lack of the genomic sequence has severely impeded research on *B. napus* miRNAs over the last years. Nevertheless, several studies, mainly based on EST/GSS sequences of *B. napus* or genomes of *B. rapa* and *B. oleracea*, have reported several hundred miRNAs and demonstrated their possible functions in regulating diverse physiological processes (He *et al.*, 2008; Korbes *et al.*, 2012; Xu *et al.*, 2012; Yu *et al.*, 2012; Zhao *et al.*, 2012; Zhou *et al.*, 2012; Huang *et al.*, 2013; Lukasik *et al.*, 2013; Wang *et al.*, 2013; Shen *et al.*, 2014). Recently, the genomes of *B. napus* and its two progenitors (A_r_A_r_ and C_o_C_o_) have been sequenced (Wang *et al.*, 2011; Chalhoub *et al.*, 2014; Liu *et al.*, 2014), providing for the first time an opportunity to identify and characterize *B. napus* miRNAs at the whole-genome level.

This work presents the results of identification and characterization of *B. napus* miRNAs by analysis of small RNA populations from two *B. napus* cultivars (one European winter cultivar,, Tapidor and one Chinese semi-winter cultivar, Ningyou7) and four double-haploid (DH) lines derived from a cross between Tapidor and Ningyou7. These data provide a first genome-wide view on the origin, evolution, and genomic organization of *B. napus MIRNA*s. Potential roles of miRNAs and their targets in the modulation of diverse physiological processes, e.g. for biosynthesis of glucosinolates and lipids, in oilseed rape are discussed.

## Materials and methods

### Plant materials

Two *B. napus* cultivars (Tapidor and Ningyou7) and their four DH lines (TN151, TN156, TN177, and TN186) (Qiu *et al.*, 2006) were used for small RNA population investigation. Tapidor and Ningyou7 have significant differences in seed erucic/glucosinolate content and flowering time. Tapidor was used to generate degradome data. The cultivar Express 617 (Shen *et al.*, 2014) was included in miRNA expression analyses. The plants were grown in a growth chamber at 24 °C and a 14h daytime photoperiod. Leaves and roots were harvested at the six-leaf stage for RNA collection. Total RNA was isolated using Trizol reagent (Invitrogen, USA). Each RNA sample was generated from leaves or roots of three different plants. The tissue samples used in RNA extraction and small RNA sequencing were collected from two replicated experiments.

### Small RNA and degradome sequencing

Small RNA libraries were constructed using the standard Illumina protocol and sequenced by Illumina HiSeq 2000 (BIOMARKER, China). Twelve libraries (two cultivars and four DH lines from two replicated experiments) were sequenced (Supplementary Table S1, available at *JXB* online). Before miRNA prediction, the adaptors and low-quality reads were removed. The clean reads were further compared with the annotated non-coding RNA sequences, including plant snoRNA (version 1.2; http://bioinf.scri.sari.ac.uk/cgi-bin/plant_snorna/home), tRNA (http://gtrnadb.ucsc.edu/), rRNA (V11.0; http://rfam.xfam.org/) and rasiRNA (release 09-02-2014; http://www.girinst.org/server/RepBase/). Two degradome libraries from the leaf and root of the six-leaf stage of Tapidor were sequenced by Illumina HiSeq 2000 (BIOMARKER) resulting in a total of 47 million raw tags. All sequences have been submitted to the GenBank/EMBL data libraries (accession no. PRJNA272953)‍.

### Prediction of miRNA and targets

The clean reads were aligned with the genome of *B. napus* (Chalhoub *et al.*, 2014; version 5.0) for identification of miRNAs. Mireap (http://sourceforge.net/projects/mireap/) was applied to predict secondary hairpin structures of *MIRNA*s with following parameters: the hairpin structure with free energy lower than –18 kcal mol^–1^; the space between miRNA and miRNA* is less than 300 nt; and with more than 16 matched nucleotides and fewer than four nucleotide bulges between miRNA and miRNA*. Only small RNAs with at least two reads in a library were used for miRNA prediction. By comparison with the miRBase (http://www.mirbase.org, release 21), a miRNA was considered as conserved if its mature sequence had two nucleotide or fewer than two nucleotide mismatches to the known miRNA or as a novel miRNA when there are more than two nucleotide mismatches (Meyers *et al.*, 2008). For the conserved miRNAs, the same miRNA/family names as in miRBase were assigned, but with new serial numbers (such as b, c, d) in some cases. For the novel miRNAs, the names bna_novel_miRX1 to bna_novel_miRX214 were given (Meyers *et al.*, 2008).

The targets of miRNAs in *B. napus* were predicted via the online sever psRNATarget (Dai and Zhao, 2011). Using the function ‘User-submitted small RNAs/ user-submitted transcripts’, the query of transcripts was generated from the *B. napus* genome. Default parameters were used to filter candidates. The software PatMan was used to map the degradomic reads to the targets, and custom perl scripts were employed to identify candidate degradative targets. According to previous results (Jones-Rhoades and Bartel, 2004; Shen *et al.*, 2014; Song et al., 2010*a*, *b*; Zhao *et al.*, 2012), the ends of degradomic reads were considered when they were within the 5 nt region of the cleavage site.

### Lipid biosynthesis-related genes

As reference, the *Arabidopsis* lipid-related gene database was downloaded at http://aralip.plantbiology.msu.edu/ (Li-Beisson *et al.*, 2013). The annotation of lipid-related genes in *B. napus* was carried out using the method of Wang *et al.* (2014) with minor modifications, i.e. BLASTp under the E-value <1e–5 and sequence identify >50% for the search of orthologous genes.

### Genomic synteny of *MIRNA*s

The synteny or co-linearity of *MIRNA*s among the three *Brassica* species (*B. napus*, *B. rapa*, and *B. oleracea*) was detected by MCScanX (Wang *et al.*, 2012). The genomes of *B. oleracea* (http://ocri-genomics.org/bolbase/, version 1.0), *B. rapa* (Phytozome 10.2) and *B. napus* (http://www.genoscope.cns.fr/brassicanapus/data/, version 5.0), and their annotated gene sets were used for the genomic synteny analysis. BLASTp was employed to determine the synteny by pairwise comparison with the parameters of E-vlaue <1e–5 and max_target_seqs < 6. *MIRNA* orthologues in *B. napus*, *B. rapa*, and *B. oleracea* were analysed using a microsynteny-based method (Ma *et al.*, 2010; Shen *et al.*, 2014). For each *MIRNA*, its 10 flanking protein-coding loci were retrieved from the *B. rapa* and *B. oleracea* genomes, respectively. Homology tests of *MIRNA* and flanking genes were performed by BLASTn and the top five hits of each *MIRNA* were chosen for flanking loci tests. A syntenic *MIRNA* pair among *B. napus*, *B. rapa*, and *B. oleracea* was defined with at least one identical upstream or downstream flanking protein-coding gene. Syntenic *MIRNA*s were divided into four sets as described by Shen *et al.* (2014). The first three sets were taken as syntenic *MIRNA*s for construction of the Circos map (Krzywinski *et al.*, 2009).

### Transcript analysis of miRNAs and target genes

Low-molecular-weight RNA was enriched by 5% polyethylene glycol (*M*_r_ 8000) and 0.5M NaCl precipitation (Lu *et al.*, 2007). Mature miRNA expression analysis was performed by stem–loop reverse transcription quantitative PCR (RT-qPCR) (Shen *et al.*, 2014). For cDNA synthesis, 200ng of low-molecular-weight RNA from each sample was reverse transcribed using a RevertAid First Strand cDNA Synthesis Kit (Thermo Scientific) with U6 snRNA as the internal control. For cDNA synthesis of predicted targets, 2 µg of total RNA from each sample was used. qPCR reactions were performed in a CFX96™ Real-Time System/C1000 Touch™ Thermal Cycler (BioRad) with MAXIMA^®^ SYBR Green Master Mix (Fermentas) and actin as the reference gene. Invariant expression of the actin gene in seedling roots and leaves was assured beforehand. The relative expression fold change of miRNAs and related genes were calculated using the comparative *C*_T_ method. All reactions were performed in triplicate with three independent experiments. The primer pairs were listed in Supplementary Table S2, available at *JXB* online.

### Expression levels of miRNAs in parental and DH lines

The expression levels of miRNAs were measured by the small RNA abundance. The amount of small RNA reads was transformed into reads per million (RPM) with the formula RPM=reads count/clean reads×10^6^). PatMan was employed to map the small RNA reads to mature miRNA sequences. Small RNA reads were counted when their position was within the length of the mature miRNA sequence. The miRNA read that was mapped to both of the A and C subgenomes was counted twice. At least a twofold change was taken to define the differentially expressed miRNAs between offspring and the mean values of parental lines in regard to the read number with corresponding mature pre-miRNAs.

### Sequences alignment and visualization

MAFFT (http://mafft.cbrc.jp/alignment/server/, version 7) was employed for multiple sequences alignment. The WebLogo (http://weblogo.threeplusone.com/create.cgi) was used to create the logo for the alignment results and CLC Sequence Viewer 6 (CLC Bio, Aarhus, Denmark) was used for the display of the conservation among whole sequences. The distribution of miRNAs and their partners was plotted in a 1Mb window size by Circos (Krzywinski *et al.*, 2009). The UEA sRNA workbench (Stocks *et al.*, 2012) was employed to visualize the hairpin structure of miRNAs with the corresponding mature sequence and parameters generated by Mireap.

### Annotation of transposable elements

As no annotation data for transposable elements in the newly released *B. napus* genome were available, transposable elements were identified in the genome using a combination of *de novo* and homology-based approaches as follows. First, the RepeatModeler pipeline was used to search for repeats in the genome and RepeatMask was then used to identify repeats in Repbase (Tempel, 2012). Long terminal repeat (LTR) retrotransposons were found using LTR_FINDER with default parameters (Xu and Wang, 2007).

### Search for miRNAs previously identified in the *B. napus* genome

BlastN was employed to map the miRNA precursor sequence onto the *B. napus* genome with an E-value of <1e–5 and a matched length longer than 90% of the query sequence.

### RNA ligase-mediated (RLM)-5′RACE analysis

To validate the predicted targets of miRNAs, 5′RACE was carried out with 2 µg of total RNA using a GeneRacer^TM^ Kit (Invitrogen) as described by Shen *et al.* (2014). The reverse transcription of mRNAs was performed using the random primers. For amplification of cDNA ends, a pair of gene-specific primers was designed (Supplementary Table S2). A touchdown PCR was performed. One microlitre of this initial touchdown PCR was used as template for the following nested PCR. The reaction products were separated on a 1.7% agarose gel. The bands with the expected size were excised, purified, and cloned into the pGEM-T vector (Promega) for sequencing.

## Results

### Identification of miRNAs

For a genome-wide analysis of *B. napus* miRNAs, 136 263 599 raw reads of 18–44 nt were generated from 12 small RNA libraries (two replicates) of young leaves of two parental lines (Tapidor and Ningyou7) and their four DH lines (Supplementary Table S1). From these, 105 219 098 clean reads were obtained. The majority of the clean reads were 21–24 nt small RNAs, consistent with a previous report (Shen *et al.*, 2014; Supplementary Fig. S1, available at *JXB* online). Of the clean reads, 64.9% could be mapped to the *B. napus* genome. After removing the non-coding RNAs, the remaining reads, referred to as endogenous small RNAs, were used for miRNA prediction.

We identified 645 miRNAs, including 280 conserved miRNAs from 34 families and 365 novel miRNAs from 225 families (Table 1 and Supplementary Table S3, available at *JXB* online). Out of 645 miRNAs, 275 and 364 originated from the A_n_ and C_n_ subgenomes, respectively (six miRNAs remained unassigned). Besides the known *B. napus* miRNAs deposited in the current international miRNA database miRBase (release 21) and reported in recent studies (Huang *et al.*, 2013; Lukasik *et al.*, 2013; Wang *et al.*, 2013; Shen *et al.*, 2014), 351 miRNAs including 47 conserved from 19 families and 304 novel miRNAs from 187 families were newly identified by this study. To confirm the expression of the predicted miRNAs, eight conserved and 14 novel miRNAs were chosen for stem–loop reverse transcription PCR analysis. The results confirmed the expected expression of all eight conserved and 13 of the 14 novel miRNAs, except for a non-specific amplification of miRX132 in *B. napus* seedlings (Supplementary Fig. S2, available at *JXB* online).

**Table 1.** miRNAs and their targets in B. napus identified in this study

Type	miRNAs		miRNAs target to:	
	Total	A_n_/C_n_	Glucosinolates^*a*^	Lipid^*a*^
Conserved	280	143/136	1 (1)	55 (27)
Novel	365	132/228	10 (6)	103 (95)
Total	645	639^*b*^	11 (7)	158 (122)

^a^ Numbers in parentheses indicate the number of glucosinolates and lipid biosynthesis-related genes that were predicted to be targeted by miRNAs.

^b^ Six miRNAs located in unplaced scaffolds.

### Prediction and validation of miRNA targets

**Fig. 1.**
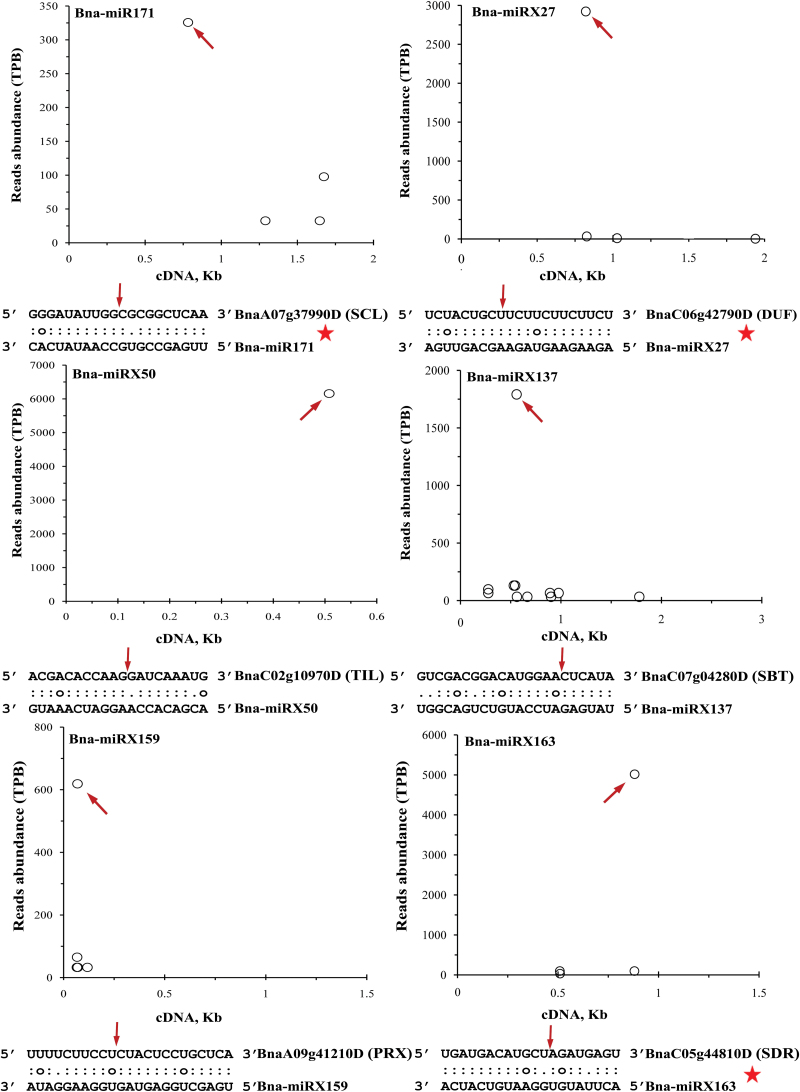
Identification of candidate targets of miRNAs by degradome experiments. Six targets of one conserved and five novel miRNAs are shown in the panels as an example. The *x*-axis indicates the position of target genes while the *y*-axis represents the abundance of sequenced reads. Each circle is a degradome fragment that can be mapped to the corresponding target gene and the arrows indicate the expected miRNA positions. The putative miRNA-binding sites are depicted within the corresponding target transcripts within the sequence alignments. The cleavage sites deduced from the degradome data are indicated by the arrows. The stars indicate the cleavages validated by the 5’RLM-RACE assays. (This figure is available in colour at *JXB* online.)

The targets of the newly identified miRNAs were predicted (Supplementary Table S4, available at *JXB* online). Consistent with the results reported previously, the majority of potential targets of the conserved miRNAs were transcription factors and shared high homology with *Arabidopsis* orthologues. For example, both miR160 and miR167 target auxin response factor (ARF) transcription factors (Mallory *et al.*, 2005; Wu *et al.*, 2006), miR156/miR157 and miR162 target squamosa promoter-binding-like protein (SPL) and Dicer-liker (DCL) protein in *Arabidopsis* and *B. napus*. Of the 365 novel miRNAs, 363 (99.4%) had putative targets in the annotated gene sets of *B. napus*. To validate the miRNA–target interaction, degradome sequencing was performed with RNA samples from Tapidor roots and young leaves, confirming 849 targets predicted for 263 conserved and 291 novel miRNAs (31 and 168 families), respectively (Fig. 1, Supplementary Table S4). Among the targets of 291 novel miRNAs, a number of genes were stress or environmental adaptation related. The *Arabidopsis* orthologue of the miRX50 target *TIL* is a heat stress-related gene (Chi *et al.*, 2009). The orthologue of the miRX137 target *SBT* is associated with the density and distribution of stomates (Berger and Altmann, 2000). miRX159 and miRX163 target the genes *PRX* and *SDR* encoding dehydrogenase/reductase and peroxidase, respectively (Passardi *et al.*, 2004; Kavanagh *et al.*, 2008).

Ten miRNAs (nine novel miRNAs) were predicted to target genes involved in biosynthesis or breakdown of glucosinolates (GSLs) (Chalhoub *et al.*, 2014) (Table 1 and Supplementary Table S5, available at *JXB* online). The miRNA-mediated regulation was confirmed for six of the 10 miRNAs either by degradomic data or by RLM-5’RACE (Supplementary Table S4, Fig. 3, Supplementary Fig. S4, available at *JXB* online). Three of these miRNAs play a role in GSL metabolism (Supplementary Table S5). 1) miRX95 was predicted to target BnaC05g12520D, an orthologue of the GSL biosynthesis-related gene *CYP79F1* (AT1G16410) in *Arabidopsis* (Chalhoub *et al.*, 2014; Liu *et al.*, 2014). The biosynthesis of GSL is mediated by *CYP79F1* (Reintanz *et al.*, 2001). There were two single-nucleotide polymorphisms between Tapidor and Ningyou7 in the predicted miRX95 target region, which resulted in additional (G:U) mismatches in the miRX95 binding site (binding prediction score >3.0) in Tapidor, which has a potential impact on the miRX95-mediated regulation of BnaC05g12520D and consequently the GSL biosynthesis in Tapidor. miR6032a was predicted to target BnaA03g38670D. Its *Arabidopsis* orthologue *APK1* (AT2G14750) is involved in the biosynthesis of a major class of sulfated secondary GSL (Mugford *et al.*, 2009). Lastly, miRX113 was predicted to target BnaA07g19610D, an orthologue of *Arabidopsis PEN3* (AT1G59870). PEN3 is required for breakdown of GSLs (Wittstock and Burow, 2010).

**Fig. 2.**
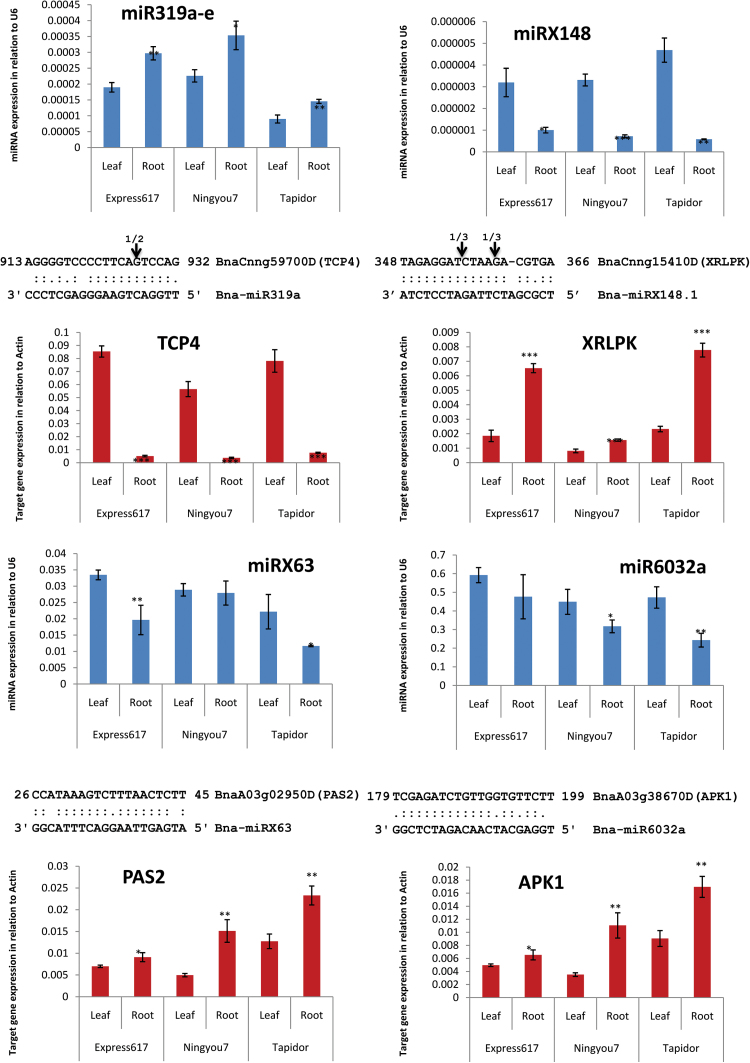
Differential expression of miRNAs and their targets in roots and leaves of three oilseed rape cultivars. The sequences depict the miRNA-binding site within the target transcript in the middle of each panel. The arrows represent the miRNA cleavage sites on targets with degradome/RACE evidence. (This figure is available in colour at *JXB* online.)

Based on a homologue search using the 738 annotated lipid biosynthesis-related genes in *Arabidopsis* (http://aralip.plantbiology.msu.edu), 2606 putative lipid biosynthesis-related genes were identified in *B. napus*. Of these, 122 genes were predicted targets of 158 miRNAs, comprising 55 conserved and 103 novel miRNAs (Table 1 and Supplementary Table S6, available at *JXB* online), from which 27 targets of 45 miRNAs were identified by the degradomic data (Supplementary Table S4). For example, one novel miRNA (miRX63) was predicted to target BnaA03g02950D, an orthologue of *Arabidopsis PAS2* or AT5G10480 that has acyl-CoA dehydratase activity and has been shown to be involved in the biosynthesis of very long chain fatty acids (see http://www.tair.org). An opposite change in the expression of miRX63 and BnaA03g02950D in leaves and roots of two of the three cultivars was obvious (Fig. 2), suggesting that miRX63-mediated regulation of the lipid biosynthesis-related gene BnaA03g02950D might play a role in the development of leaf and root. As GSL and lipid biosynthesis is regulated by complex mechanisms and by developmental stages (Grubb and Abel, 2006; Pollard *et al.*, 2008; Niu *et al.*, 2009), more detailed investigations are needed to clarify the role of these miRNA–target interactions in the biosynthesis of GSL and lipids in the future.

To identify miRNAs involved in regulating different physiological processes in roots and leaves, respectively, the small RNA populations from leaves were compared with those from roots of *B. napus* (Shen *et al.*, 2014) and 59 miRNAs were identified including 35 novel ones, which gave a significant difference in the read numbers (>2-fold) between the roots and leaves (Supplementary Table S7, available at *JXB* online). Differential expression was confirmed for 10 selected miRNAs (four conserved and six novel miRNAs) by stem–loop qPCR (Supplementary Fig. S3, available at *JXB* online). Among these, several miRNAs (such as miR159 and miR319) have a pronounced role in regulating plant leaf/root development (Khraiwesh *et al.*, 2012).

The expression profiles of four of these miRNAs and their targets in roots and leaves were further investigated using RT-qPCR. As shown in Fig. 2, in all four cases (miR319, miR6032, miRX63, and miR148), a reciprocal change in the expression levels between the miRNA and its target was evident. Thus, these data suggested a direct miRNA–target expression modulation in regulation of developmental and/or physiological processes in the root and leaf.

### Evolution of miRNAs after the allopolyploidization event

**Fig. 3.**
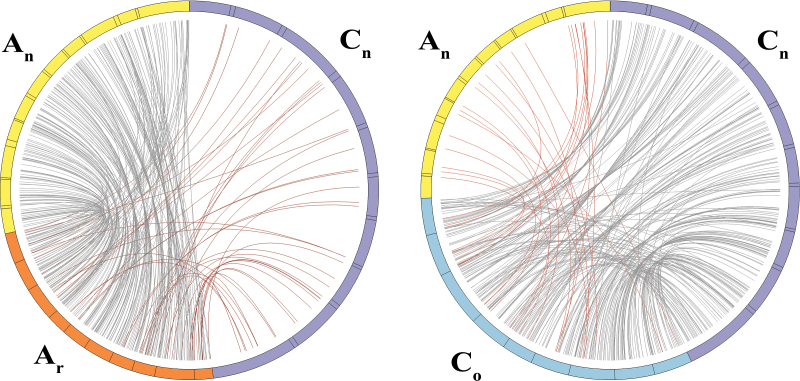
Synteny of miRNAs between *B. napus* (A_n_A_n_C_n_C_n_) and its two progenitor genomes (A_r_A_r_ and C_o_C_o_). The 851 *MIRNA*s from the *B. rapa* and *B. oleracea* genomes were mapped to the *B. napus* genome. The lines refer to the best matches of the miRNAs from the two progenitors in the *B. napus* genome. A_n_ and C_n_ represents the A and C subgenome of *B. napus*, A_r_ indicates the genome of *B. rapa*, and C_o_ stands for the genome of *B. oleracea.* The black lines indicate the miRNA best match in A_n_-A_r_ and C_n_-C_o_, and the red lines represent the miRNA best match in A_n_-C_o_ and C_n_-A_r_.

**Fig. 4.**
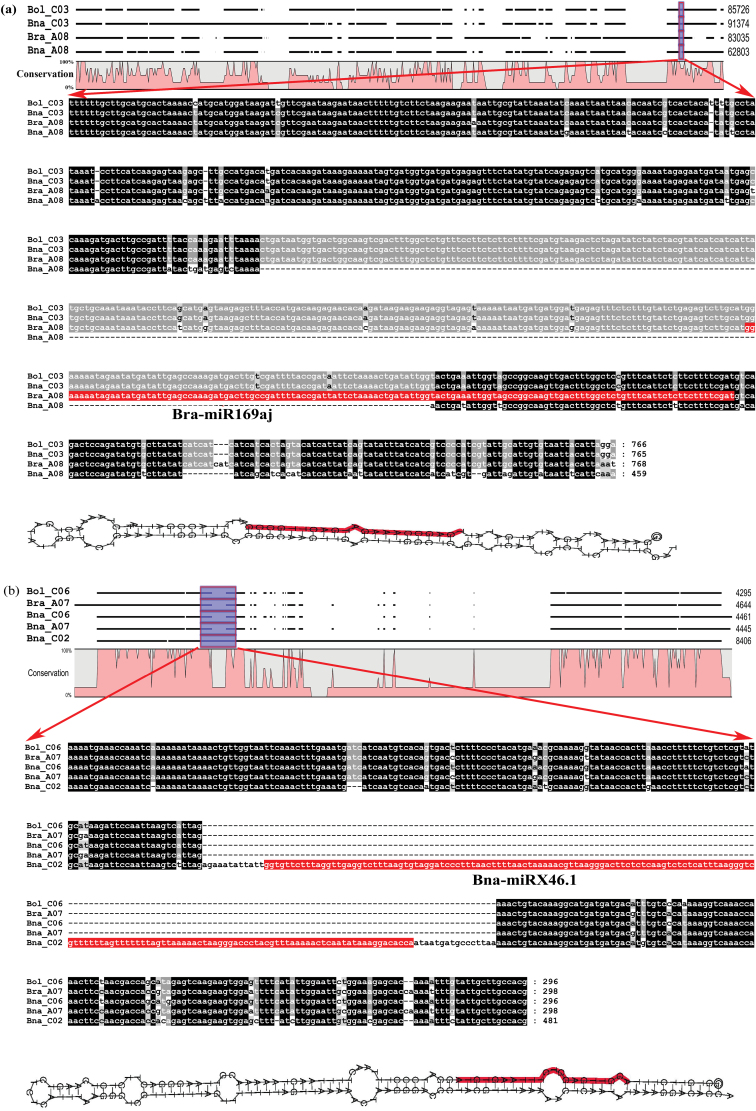
Gain and loss of miRNAs in *B. napus*. (a) An example of miR169 loss in *B. napus*. The upper part was the synteny region among the three species. The deletion of seveval bases on chromosome A08 of *B. napus*, which caused the loss of one member of miR169 family in *B. napus*. (b) The novel-miRX46.1 as example for the gain of miRNAs in *B. napus*. The upper part indicates the synteny region found by MCScan, in which an insertion sequence was detected on the chromosome C02 of *B. napus*. The sequence can form a perfect hairpin structure as indicated in the lower part.

The availability of the genomes of *B. napus* and their two progenitors provides an opportunity to look at the expansion and loss of miRNAs in *B. napus*. The synteny of *MIRNA*s between *B. napus* (A_n_A_n_C_n_C_n_) and its two progenitor genomes (A_r_A_r_ and C_o_C_o_) was first investigated by mapping the 851 *MIRNA*s identified from the two progenitor genomes in a previous study (Shen *et al.*, 2014) to the *B. napus* genome. To ascertain genomic synteny of the predicted *MIRNA*s between the genomes of *B. napus* and its two progenitors, a microsynteny analysis was performed using the *MIRNA*s and their flanking protein-coding sequences. As expected, the majority (646/851, 75.9%) of the two progenitor *MIRNA* orthologues existed in the corresponding subgenomes of *B. napus* (Supplementary Table S8, available at *JXB* online), although a small number (72) of these *MIRNA*s showed a match to the opposite subgenome of *B. napus* (Fig. 3). These results support the observation of a high conservation between the subgenomes of *B. napus* and its two progenitor genomes (Chalhoub *et al.*, 2014). In addition, 115 *MIRNA*s, including 104 novel *MIRNA*s, failed to show any homologous sequence hit in the *B. napus* genome (Supplementary Table S9, available at *JXB* online). Of these, five conserved *MIRNA*s (e.g. members of the miR169 and miR172 families) were obviously lost due to sequence deletions or mutations as revealed by comparative genome analysis between *B. napus* and it two progenitors (Table 2; an example of miR169 member is shown in Fig. 4a).

**Table 2.** Changes of copy numbers of miRNA families between B. napus and its two progenitors (B. rapa and B. oleracea) Only part of the miRNA families are shown here and a full list is provided in Supplementary Table S10, available at *JXB* online.

Family	*B. napus*		*B. rapa* ^*a*^	B. oleracea^*a*^	(A_n_A_n_C_n_C_n_)-(A_r_A_r_+C_o_C_o_)	
	A_n_A_n_	C_n_C_n_	A_r_A_r_	C_o_C_o_	Based on small RNAs	Based on genomic synteny
miR156	17	19	17	15	4	5
miR171	11	7	7	3	8	3
miR166	13	11	6	4	14	2
miR168	9	9	5	7	6	1
miR169	16	15	26	19	–14	–2
miR172	8	6	11	9	–6	–2
miR390	6	2	6	6	–4	–1
miR395	5	5	9	6	–5	0
miR159	1	2	4	4	–5	0
miR398	1	1	4	3	–5	0
miR160	9	9	7	7	4	0
miR165	2	0	3	3	–4	0

^a^ Based on Shen *et al.* (2014).

In the next step, the 645 miRNAs of *B. napus* were compared with *Arabidopsis* miRNAs (miRBase, release 21). A significant expansion of several miRNA families, including miR156, miR160, and miR169 in *B. napus*, was observed (Supplementary Table S10). Since such expanded miRNA families could also be found in the two progenitor genomes, it was concluded that these expansion events must have occurred before the AA-CC speciation hybridization (Supplementary Table S10).

However, 18 *B. napus MIRNA*s (six novel and 12 conserved *MIRNA*s) were not present in the syntenic regions of the two progenitor genomes, suggesting that they might be newly generated after the allopolyploidization event in *B. napus* (Supplementary Table S11, available at *JXB* online). The 12 newly generated conserved *MIRNA*s, including six, two, and three from the miR156, miR171, and miR166 families, respectively (Table 2 and Supplementary Table S11), obviously originated from the existing *MIRNA*s via the gene or genomic segmental duplication events. The expression of three of the six novel miRNAs (bna-miRX46 as an example shown in Fig. 4b) could be confirmed in *B. napus* seedlings by the stem–loop reverse transcription PCR analysis (Supplementary Fig. S2). The predicted targets of the six novel miRNAs could be confirmed by the degradome sequencing (Supplementary Table S4). These newly evolved *B. napus* miRNAs might be associated with adaptation to changed environmental conditions under natural and artificial selection pressure. In support of this, the targets of miRX132.1 (BnaA05g30540D for the putative defensin-like protein 73 and BnaA03g50380D for the putative F-box/LRR-repeat protein 19) were related to plant defence responses. Also, the target of miR46.1 (BnaC03g35590D for phosphatidylinositol 4-phosphate 5-kinase 6) interacts with the lipid pathway (Supplementary Table S8).

### Partitioning and heritability of miRNA expression

**Fig. 5.**
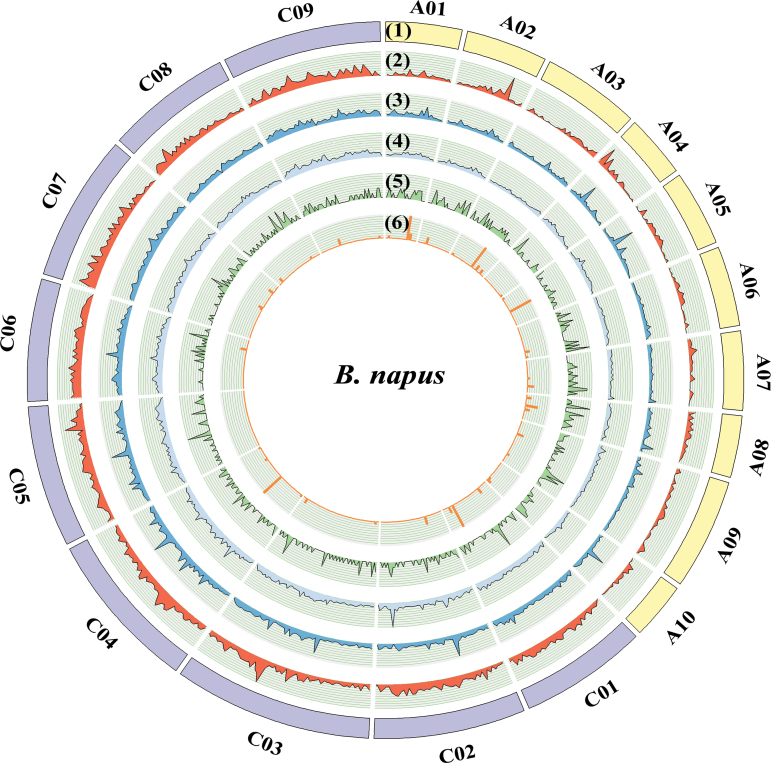
Genomic distribution of miRNAs in *B. napus*. From outer to inter circles: (1) chromosomes of *B. napus*; (2) LTR retrotransposon; (3) small RNA reads; (4) 24 nt small RNA reads; (5) miRNAs identified by this study; (6) small RNA reads that can be mapped to the mature sequences of miRNAs. The peak value represents the density of each element with a window of 1Mb in the *B. napus* genome.

To investigate the partitioned expression of miRNAs between the A_n_ and C_n_ subgenomes, the expression levels and patterns of miRNAs in the A_n_ or C_n_ subgenome were compared based on small RNA reads. Although 275 and 364 *MIRNA*s were identified from the A_n_ and C_n_ subgenomes, respectively, a slightly higher genomic density of *MIRNA*s was observed in the A_n_ subgenome (1.03 and 0.73 *MIRNA*s per Mb for the A_n_ and C_n_ subgenomes), which was similar to those observed in the two progenitor genomes (Shen *et al.*, 2014). To measure miRNA expression (see Materials and methods), the number of small RNA reads of corresponding mature miRNAs were compared, and a significantly higher abundance of reads (per unique mature miRNA sequence per million reads) was observed in the A_n_ subgenome than in the C_n_ subgenome (*P*<0.01) (Table 3, Fig. 5). To confirm this, small RNA populations were generated from Tapidor and Ningyou7 seedlings grown under independent environmental conditions and sequenced. A similar bias of miRNA expression was obvious between the two subgenomes (Table 3). The comparison of miRNA expression patterns of four Tapidor/Ningyou7-derived DH lines with those of their parental lines also gave similar biased expression patterns of miRNAs (*P*<0.01 and *P*<0.02 in two environments, respectively; Table 3). When the individual miRNA families (for 32 with members > 3) were compared, only 13 of the 32 families were found, which were the main contributors to the different miRNA read abundance or miRNA expression levels between the A_n_ and C_n_ subgenomes, while the remaining 19 miRNA families showed a similar expression pattern between the A_n_ and C_n_ subgenomes. These data strongly suggested a partitioned expression of miRNA between the two subgenomes after the hybridization of speciation in *B. napus*.

**Table 3.** Comparison of miRNA expression in the two subgenomes in B. napus The expression levels were measured by Illumina sequencing reads per unique mature sequence of miRNA or per Mb per million reads in two DH parental lines (Tapidor and Ningyou7 or T/N) and their four DH lines.

Expression level (read density)^*a*^	A_n_	C_n_	C_n_/A_n_	*P* value	Based on:
Reads (E1) per mature miRNA	4293.26	2360.90	0.55	<0.01	T/N
Reads (E2) per mature miRNA	3119.39	2261.51	0.72	<0.01	T/N
Reads (E1) per Mb	3928	1493	0.52	<0.01	T/N
Reads (E2) per Mb	2854	4231.09	0.68	<0.01	T/N
Reads (E1) per mature miRNA	6226.15	4231.09	0.68	<0.01	4 DH lines
Reads (E2) per mature miRNA	2006.64	1449.13	0.72	0.02	4 DH lines
Reads (E1) per Mb	5696.26	2792.52	0.49	<0.01	4 DH lines
Reads (E2) per Mb	1835.86	956.43	0.52	<0.01	4 DH lines

^a^ Small RNA populations in two parental lines (T/N) were collected and sequenced in two different environments (E1 and E2), respectively.

To investigate the heredity of miRNA expression, small RNA populations from seedlings of the same developmental stage (six leaves) of the two parental lines (Tapidor and Ningyou7) and their four DH lines were analysed. After normalization of small RNA reads, the expression levels of miRNAs in Tapidor and Ningyou7 were compared with those in the four DH lines. Using a 2-fold change of the average expression level of a miRNA in the four DH lines relative to their parental lines as the threshold value (Stupar *et al.*, 2007), the additive genetic effect of miRNAs in *B. napus* was estimated. Significantly, a higher number of miRNAs with a non-additive rather than with an additive genetic effect (2- to 3-fold) were observed in the four DH lines (Supplementary Table S12, available at *JXB* online), suggesting the potential dominance of non-additive genetics of miRNAs in *B. napus*.

Finally, the expression levels of other small RNAs in the *B. napus* genome were investigated and a genomic bias was also observed for siRNA expression (Fig. 5). However, this was totally in contrast to the A_n_-biased miRNA expression, i.e. a higher abundance of small RNA reads was observed in the C_n_ but not in the A_n_ subgenome (2.1-fold difference between the two subgenomes), suggesting that more siRNAs are generated or expressed from the C_n_ subgenome. Consistent with this, the content of LTR retrotransposons was found to be 10.3-fold higher in the C_n_ subgenome than in the A_n_ subgenome (Fig. 5), a phenomenon also observed previously (Chalhoub *et al.*, 2014; Liu *et al.*, 2014), implying the possible involvement of these repetitive transposable elements in the biogenesis of siRNAs.

## Discussion

This study carried out the first genome-wide investigation of miRNAs in *B. napus* using the newly sequenced *B. napus* genome. In addition to known *B. napus* miRNAs, identified mainly based on the two progenitor genomes of *B. napus* (Zhou *et al.*, 2012; Korbes *et al.*, 2012; Xu *et al.*, 2012; Zhao *et al.*, 2012, Shen *et al.*, 2014), a new set of *B. napus* miRNAs was identified, including both conserved and novel ones. As expected, the number of *B. napus* miRNAs identified so far is significantly higher than that in the model dicot species *Arabidopsis thaliana* and monocot species rice (*Oryza sativa*). To the best of our knowledge, this represents the largest miRNA population ever found in a single plant species up to now, thus offering a unique dataset for the study of the origin, genomic structure, and evolution of miRNAs in allopolyploid plant genomes.

The results of this study support the observation of a high genomic conservation between the subgenomes of *B. napus* and its two progenitor genomes (Chalhoub *et al.*, 2014). In many cases, it was not possible to distinguish the miRNA reads between the A and C subgenomes as they share the same mature sequence. The genomic synteny regions applied in this study allowed a precise localization of miRNA loci in the orthologous regions of both subgenomes. Only miRNAs with unique mature sequences in the A or C subgenome were defined as A or C subgenome-specific sequences, respectively. The majority of the two progenitor *MIRNA* orthologues were found to exist in the corresponding subgenomes of *B. napus*, and only a small group of *MIRNA*s showed an opposite match to the subgenomes. These mismatches might be a result of the homeologous recombination or gene-level duplication events during/after the AA-CC hybridization. Also, 115 *MIRNA*s were found only in the genomes of the two progenitors and not in the *B. napus* genome, strongly suggesting a possible loss of these *MIRNA*s due to sequence deletion or mutation (Table 2, Fig. 4a), although these *MIRNA*s could be undetected due to limited assembly of the two progenitor genomes. Because of a high genetic redundancy after the AA-CC hybridization, it is conceivable that the miRNA loss occurred in the *B. napus* genome. As *B. napus* has a very recent origin (~7500 years ago; Chalhoub *et al.*, 2014), it would be expected that the numbers of conserved miRNAs in *B. napus* will be decreased to a relatively stable level in future, as observed in the tetraploid cotton genome (Xie and Zhang, 2015). However, the allopolyploidization event resulted in an increase in the number of *MIRNA*s in *B. napus* by generating new *MIRNA*s through gene or genomic segmental duplication events. As revealed by the genomic microsynteny analysis, at least 12 of the 18 newly generated *MIRNA*s in *B. napus* obviously originated from the existing conserved *MIRNA*s (miR156, miR171, and miR166 families) in the two progenitors via such genomic reorganization. For instance, miR156 affects the flowering time and responses to biotic and abiotic stress (Sunkar *et al.*, 2012; Stief *et al.*, 2014). Similarly, miR171 also participates in the biotic and abiotic stress responses (Zhou *et al.*, 2007; Liu *et al.*, 2008), while miR166 can alter the development and polarity of the leaf via an interaction with HD-ZIP genes (Emery *et al.*, 2003; Williams *et al.*, 2005). Following this, it is reasonable to speculate that the 12 new copies of these conserved *MIRNA*s might greatly contribute to the environmental adaptation of *B. napus.* Their origins might be attributed to natural selection. In addition, a significant expansion of a few conserved miRNA families, such as miR156, miR160, and miR169, have obviously enlarged the number of miRNAs in *B. napus* (Supplementary Table S10), but these expansions have mostly occurred before the AA-CC hybridization, as such expansion events could also be observed in the two progenitor genomes (Supplementary Table S10). This is consistent with the very recent origin of the AACC genome in *B. napus*. Unequal genomic/segmental duplications as well as tandem duplications of *MIRNA*s obviously might also have greatly contributed to the *MIRNA* expansion in the *B. napus* genome (Shen *et al.*, 2014).

An interesting finding in this study was the partitioned expression of miRNAs between the two subgenomes of *B. napus*. A significantly higher abundance of miRNAs in the A_n_ subgenome than in the C_n_ subgenome was observed in at least 13 miRNA families in two different genetic backgrounds (Tapidor and Ningyou7) and under different environmental conditions. Furthermore, similar results observed in the four Tapidor/Ningyou7-derived DH lines provide further genetic evidence for partitioning of miRNA expression between the two subgenomes in *B. napus.* It is of great interest and importance to explore the regulation and functional relevance of the partitioning of miRNA expression in oilseed rape in the future. In this study, a potential dominance of non-additive genetics for miRNAs in *B. napus* was suggested for the first time by heredity analyses of the expression patterns of each miRNA using small RNA populations from the parental lines (Tapidor and Ningyou7) and four Tapidor/Ningyou7-derived DH lines. Nevertheless, further investigations using more DH lines or offspring lines from different parental lines and in particular their degradome data are needed to confirm this observation.

miRNAs are believed to have played an important role in genome polyploidy (Ng *et al.*, 2012), as well as in regulating network in speciation and subsequent evolution of polyploidy crops (Yao *et al.*, 2007; Kenan-Eichler *et al.*, 2011; Tang *et al.*, 2012; Gong *et al.*, 2013; Xie and Zhang, 2015). In upland cotton (AADD, *Gossypium hirsutum* L.), miRNA loss and gain after the hybridization of AA and DD species were detected at the genomic level. However, significant expansions of several AADD-specific miRNA families were identified in cotton but not in *B. napus*. This might be attributed to the post-Neolithic origin of *B. napus* (Chalhoub *et al.*, 2014). The allotetraploid cotton arose from the union of two types of diploid cotton genomes about 1–2 million years ago (Li *et al.*, 2015; Zhang *et al.*, 2015). Similar to the observations here in *B. napus*, the biased expressions of miRNAs in the two subgenomes of a tetraploid were also observed in cotton (Gong *et al.*, 2013; Xie and Zhang, 2015). Significantly higher expression of ~20 conserved miRNAs in the AA subgenome than in the DD subgenome in cotton were reported (Gong *et al.*, 2013). These results suggest that miRNAs play an important role in the regulation network in speciation and subsequent evolution of polyploidy crops.

Increasing data suggest that miRNAs are involved in regulating plant responses to diverse developmental and physiological processes, as well as plant responses to diverse biotic and abiotic stress (Khraiwesh *et al.*, 2012). By comparison with previously sequenced small RNA populations from roots (Shen *et al.*, 2014), a number of miRNAs were identified here that are differentially expressed between the root and leaf of *B. napus*. Among these are miR159, miR164, miR166, miR168, miR172, miR319, and miR390, which have been demonstrated previously to have an effect on leaf development in *Arabidopsis* (Emery *et al.*, 2003; Palatnik *et al.*, 2003; Achard *et al.*, 2004; Allen *et al.*, 2005; Laufs *et al.*, 2004; Vaucheret *et al.*, 2004; Guo *et al.*, 2005; Schwab *et al.*, 2005; Williams *et al.*, 2005), as well as miR160 and miR393, which have been shown to influence the development of roots via inhibition of their target genes (*ARF* and *TIR*, respectively) in *Arabidopsis* (Navarro *et al.*, 2006; Liu *et al.*, 2007). The opposite expression patterns between miRNAs and their targets occurred in the root and leaf of *B. napus*, suggesting their conserved role in the regulation of developmental or physiological processes in the root and leaf of *B. napus.* Of great importance, a subset of novel miRNAs were identified that could have a specific function in regulating the expression of specific traits, such as biosynthesis of lipids and GSLs in oilseed rape. Further experimental investigation and analysis are needed to gain insights into the underlying mechanisms.

## Supplementary data

Supplementary data are available at *JXB* online.

Supplementary Table S1. Small RNAs generated from *B. napus* by this study.

Supplementary Table S2. Primers used in this study.

Supplementary Table S3. A list of miRNAs identified in *B. napus* by this study.

Supplementary Table S4. Predicted miRNA targets and targets with degradomic evidence.

Supplementary Table S5. Details about miRNAs predicted to target glucosinolate-related genes in Darmor (reference genome), Tapidor, and Ningyou7.

Supplementary Table S6. Lipid-related miRNAs identified in *B. napus* in this study.

Supplementary Table S7. Differential expressed miRNAs in root and leaf at the seedling stage.

Supplementary Table S8. The 646 miRNAs (Shen *et al.*, 2014) with high similarity (>95%) between *B. napus* and its two progenitors.

Supplementary Table S9. The 851 miRNAs previously identified in the two progenitor genomes (Shen *et al.*, 2014) mapped to the *B. napus* genome.

Supplementary Table S10. Changes of conserved miRNA family sizes in *B. napus* and its two progenitors compared with *A. thaliana.*

Supplementary Table S11. The loss and gain of miRNAs in *B. napus* after the hybridization of its two progenitors.

Supplementary Table S12. The additive genetic effects of miRNA expression between DH lines and their parents.

Supplementary Fig. S1. Frequency distribution of small RNA reads with different length by this study.

Supplementary Fig. S2. Expression analysis of nine conserved and 13 novel miRNAs by stem–loop RT-PCR.

Supplementary Fig. S3. Eleven differentially expressed miRNAs in leaf and roots in three *B. napus* cultivars.

Supplementary Fig. S4. RACE reactions with three selected miRNAs as examples.

Supplementary Data
