# Supplementary material for: Identification, evolution, and expression partitioning of miRNAs in allopolyploid Brassica napus
Source: J Exp Bot. 2015 Sep 10;66(22):7241–53. doi: 10.1093/jxb/erv420 (PMC4765792; doi:10.1093/jxb/erv420)
Supplement: Supplementary Data [file supp_66_22_7241__index.html]

Identification, evolution, and expression partitioning of miRNAs in allopolyploid Brassica napus — Supplementary Data 

# Identification, evolution, and expression partitioning of miRNAs in allopolyploid *Brassica napus*

## Supplementary Data

Data files

- Supplementary\_FigureS1\_S4.pdf - Supplementary Data
- Supplementary\_Table\_S1.xls - Supplementary Data
- Supplementary\_Table\_S2.pdf - Supplementary Data
- Supplementary\_Table\_S3.xls - Supplementary Data
- Supplementary\_Table\_S4.xls - Supplementary Data
- Supplementary\_Table\_S5.xls - Supplementary Data
- Supplementary\_Table\_S6.xls - Supplementary Data
- Supplementary\_Table\_S7.xlsx - Supplementary Data
- Supplementary\_Table\_S8.xls - Supplementary Data
- Supplementary\_Table\_S9.xls - Supplementary Data
- Supplementary\_Table\_S10.xlsx - Supplementary Data
- Supplementary\_Table\_S11.xls - Supplementary Data
- Supplementary\_Table\_S12.xls - Supplementary Data
