# Supplementary material for: Identification, evolution, and expression partitioning of miRNAs in allopolyploid Brassica napus
Source: J Exp Bot. 2015 Sep 10;66(22):7241–53. doi: 10.1093/jxb/erv420 (PMC4765792; doi:10.1093/jxb/erv420)
Supplement: Supplementary Data [file supp_erv420_Supplementary_FigureS1_S4.pdf]

Identification, evolution and expression partitioning of miRNAs in allopolyploid *Brassica napus*

Enhui Shen, Jun Zou, Falk Behrens, Li Chen, Chuyu Ye, Shutao Dai, Ruiyan Li, Meng Ni, Xiaoxue Jiang, Jie Qiu, Yang Liu, Weidi Wang, Qian-Hao Zhu, Boulos Chalhoub, Ian Bancroft, Jinling Meng, Daguang Cai, Longjiang Fan

Fig. S1 Frequency distribution of small RNA reads with different length by this study

Fig. S2 Expression analysis of 9 conserved and 13 novel miRNAs by stem-loop RT-PCR

Fig. S3 Eleven differentially expressed miRNAs in leaf and roots in three *B. napus* cultivars

Fig. S4 RACE reactions with 3 selected miRNAs as examples

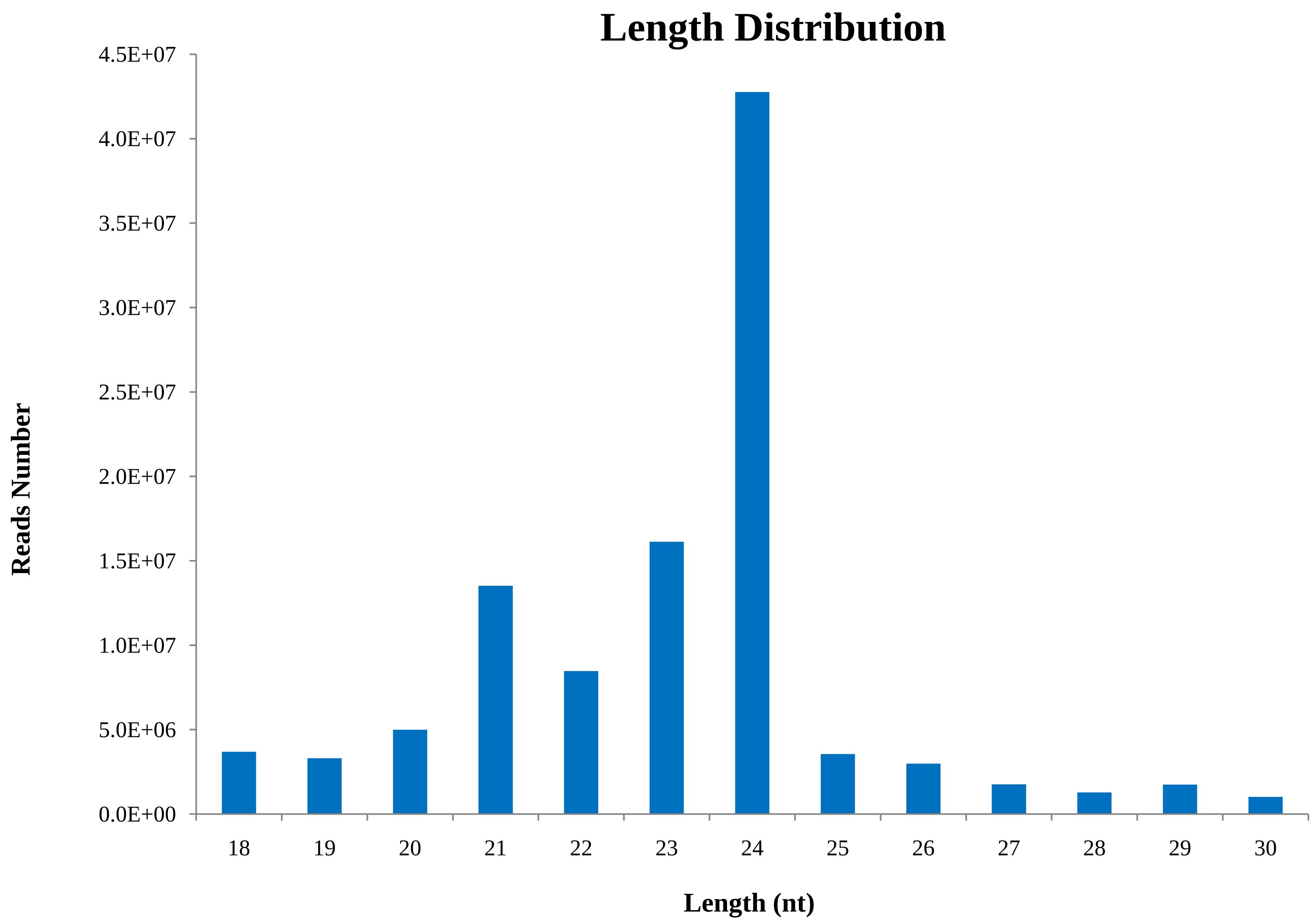

Fig. S1 Frequency distribution of small RNA reads with different length by this study

Number	miRNA	Mature Sequences(5'-3')
1	miR156s-v	TTGACAGAAGAAAGAGAGCAC
2	miR171n_3p	TGATTGAGCCGCGCCAATATC
3	miRX92	CTTACTTGTAAGTCGTCTGTG
4	miRX123	GTTTAGCTGTAGATAAATCGGTT
5	miRX209	TTTCGTTCTTGTGGGAAGCAT
6	miRX132	TAGAGGTGGGTTGGAGATTGGT
7	miRX13	AATCGGGGGTTGACGCTTCAG
8	miRX46	AGGTTGAGGTCTTTAAGTGTA
9	miR169b	CAGCCAAGGATGACTTGCCGG
10	miR397a-d	TCATTGAGTGCAGCGTTGATGT
11	miRX205	TTGTAGAATTTTGGGAAGGGC
12	miR159a-c	TTTGGATTGAAGGGAGCTCTA
13	miR319a-e	TTGGACTGAAGGGAGCTCCCT
14	miR827a-d	TTAGATGACCATCAACAAATA
15	miR845a;b	CGGCTCTGATACCAATTGATG
16	miRX136	TATCTACTGCTTATGCCACCA
17	miRX148.1	TCGCGATCTTAGATCCTCTAA
18	miRX63	ATGAGTTAAGGACTTTACGGA
19	miRX95	CTTGTCGGAGTTTATGATCTC
20	miRX210	TTTCTCGGAAATTCTTGCATA
21	miR6032a	TGGAGCATCAACAGATCTCGG
22	miRX113	GGGACTCGAACTCGTGATGGC

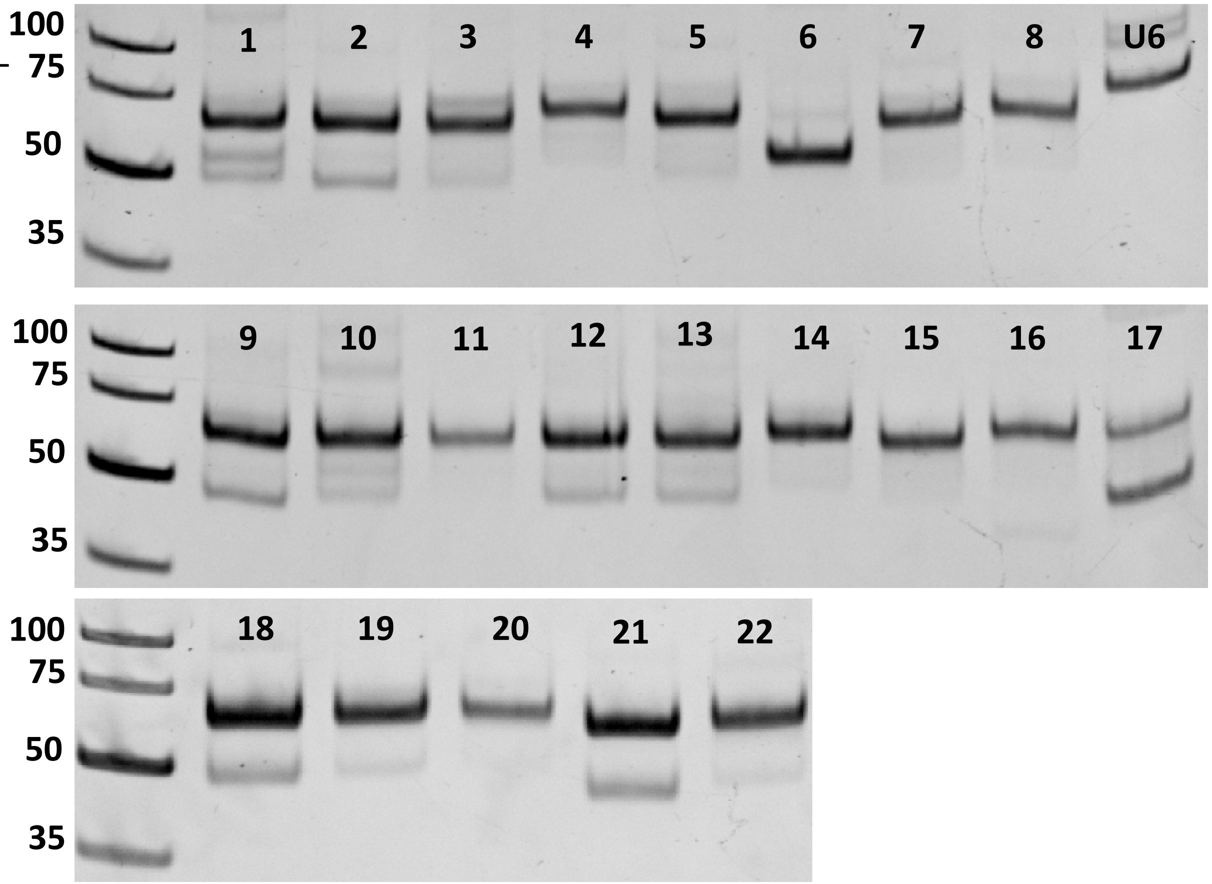

Fig. S2 Expression analysis of 9 conserved and 13 novel miRNAs by stem-loop RT-PCR

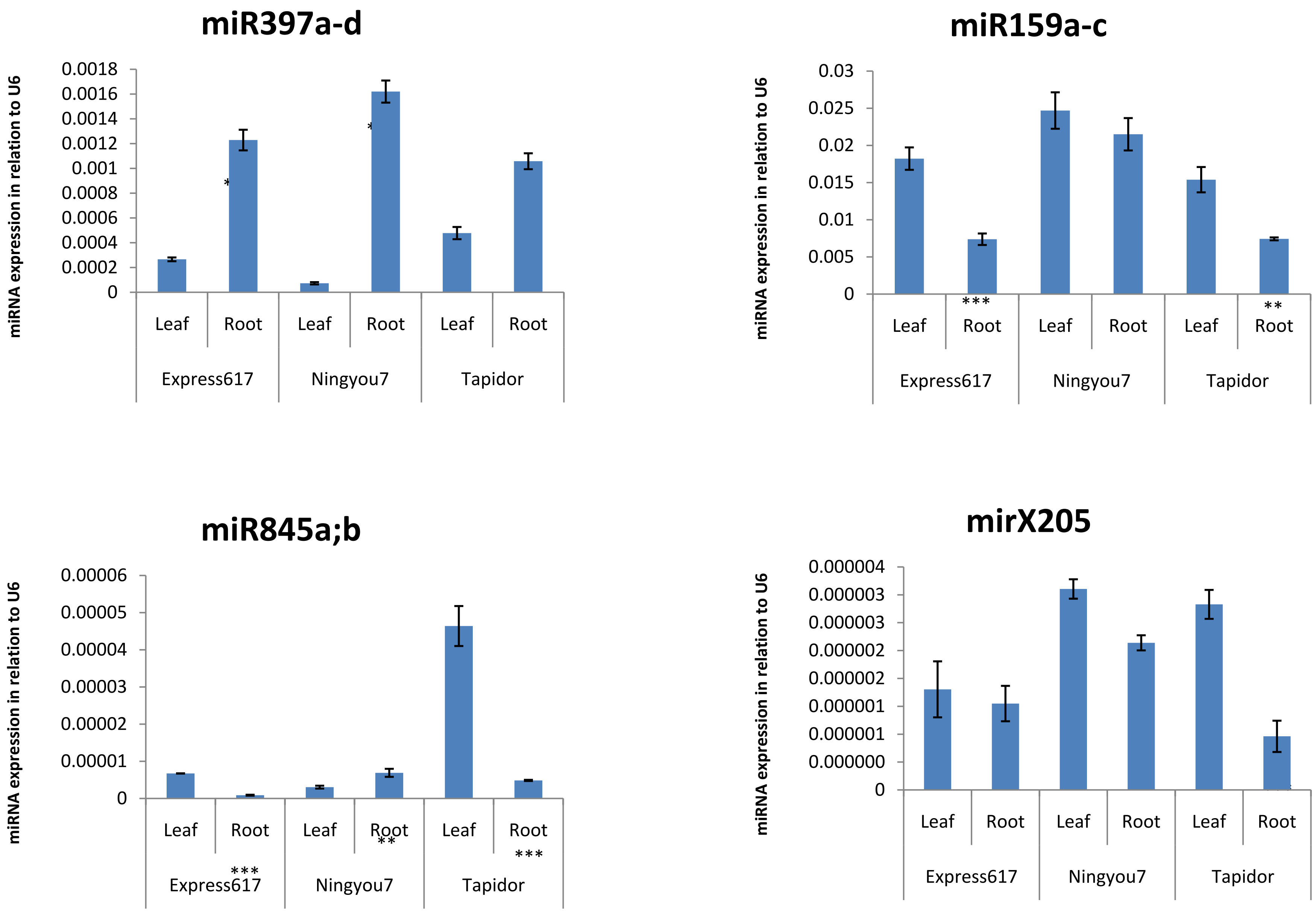

Fig. S3a Eleven differentially expressed miRNAs in leaf and roots in three *B. napus* cultivars

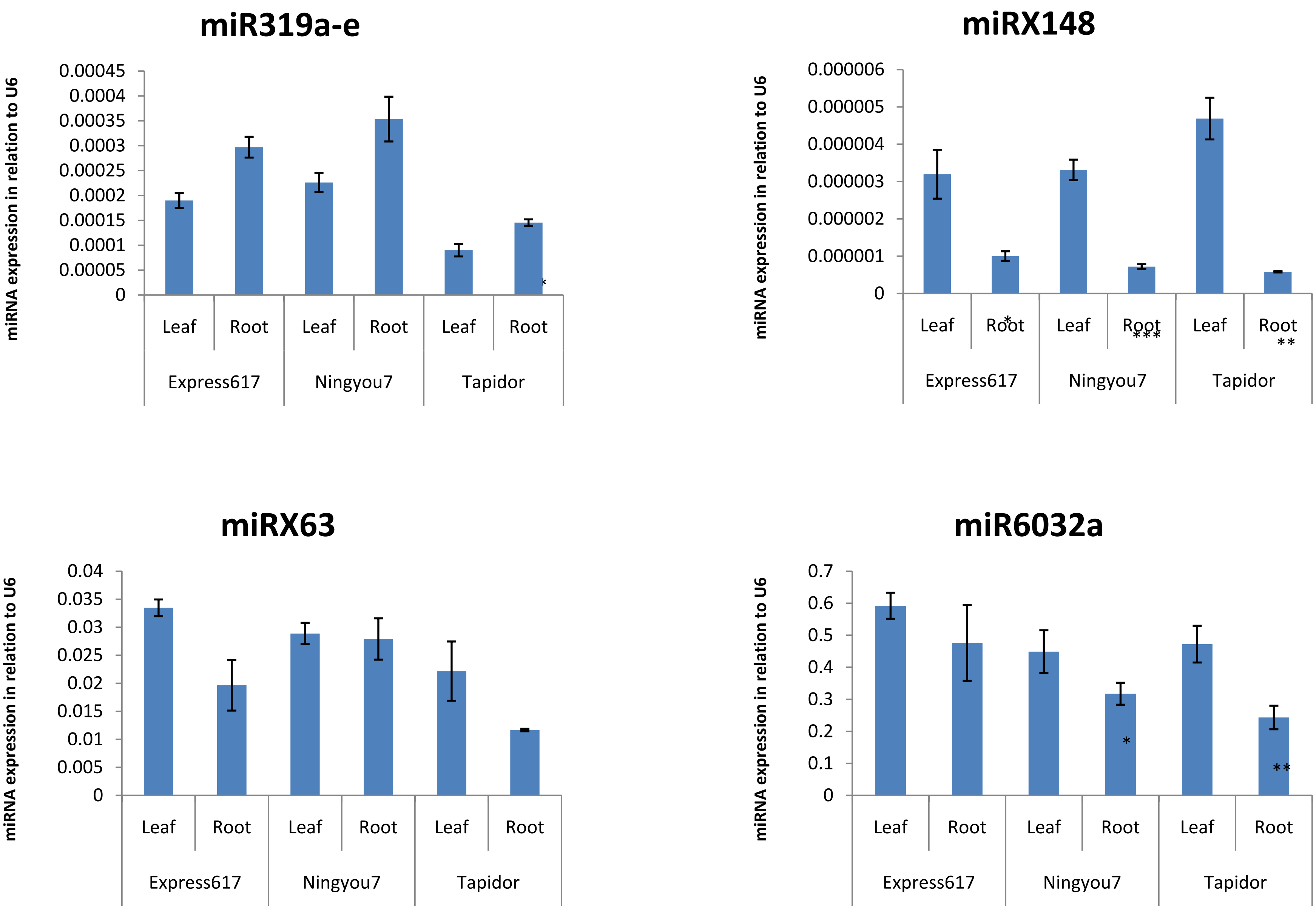

Fig. S3b Eleven differentially expressed miRNAs in leaf and roots in three *B. napus* cultivars

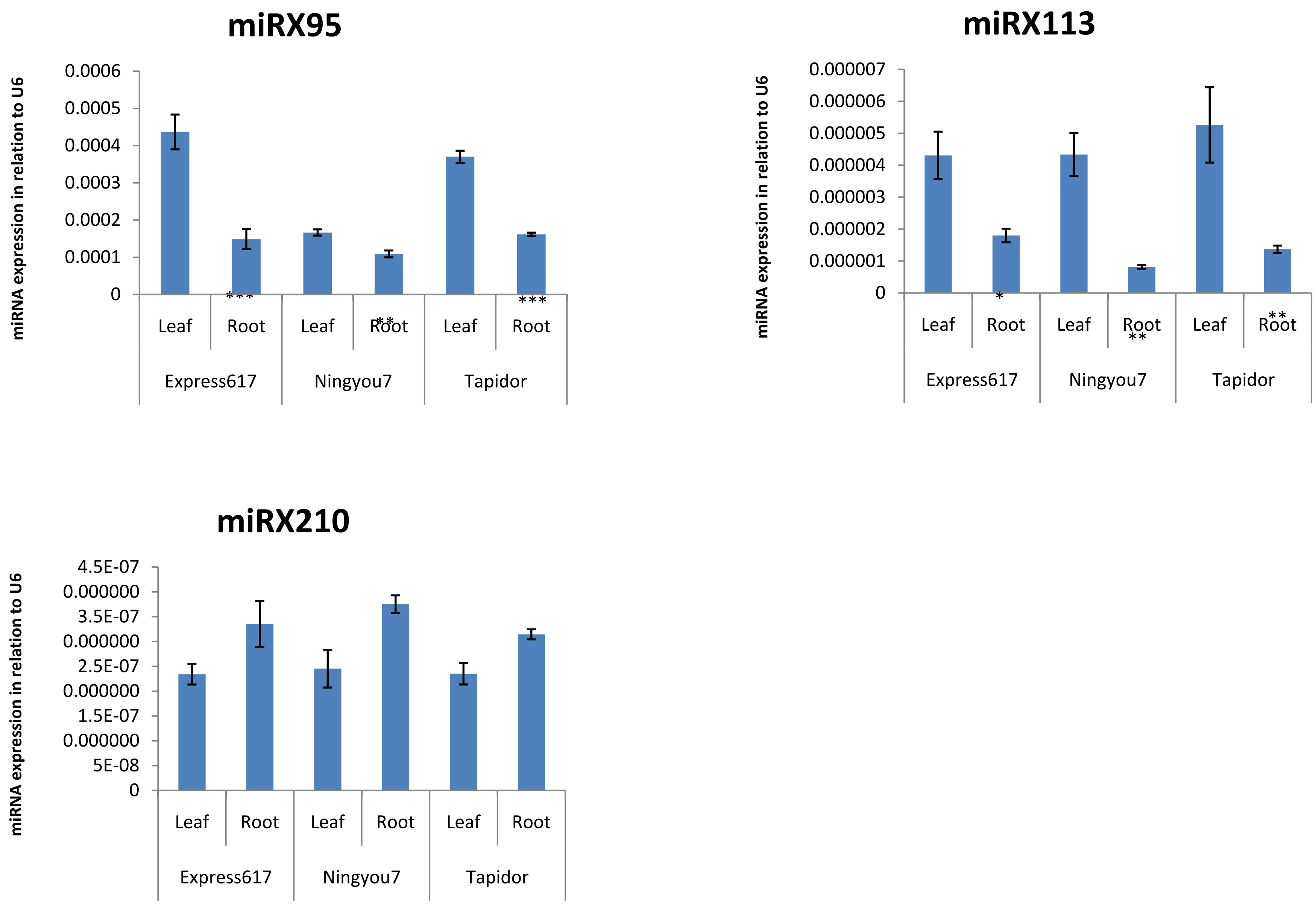

Fig. S3c Eleven differentially expressed miRNAs in leaf and roots in three *B. napus* cultivars

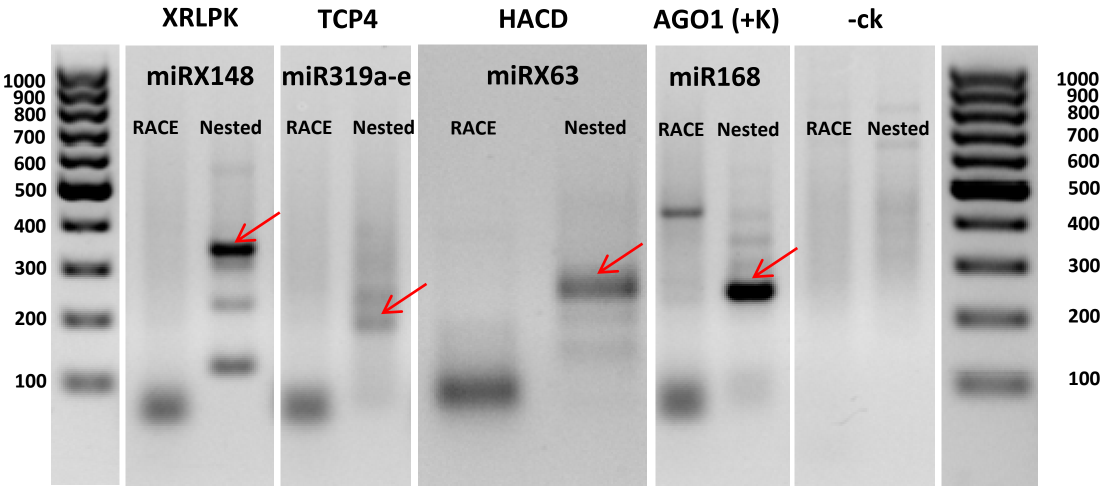

Red arrow indicates expected band

Fig. S4 RACE reactions with 3 selected miRNAs as examples
