# Supplementary material for: Identification, evolution, and expression partitioning of miRNAs in allopolyploid Brassica napus
Source: J Exp Bot. 2015 Sep 10;66(22):7241–53. doi: 10.1093/jxb/erv420 (PMC4765792; doi:10.1093/jxb/erv420)
Supplement: Supplementary Data [file supp_erv420_Supplementary_Table_S2.pdf]

Supplementary Table S2

a) miRNA expression primer

miRNA	RT-primer sequence (5'-3')	Forward primer sequence (5'-3')
miR156s-v	N44GTGCTC	GCGCTTGACAGAAGAAAGAGAGC
miR171n_3p	N44GATATT	GCTGATTGAGCCGCGCCAA
miRX92	N44CACAGA	GCGCCTTACTTGTAAGTCGTCTG
miRX123	N44AACCGA	GCGCGTTTAGCTGTAGATAAATCG
miRX209	N44ATGCTT	GCGCTTTCGTTCTTGTGGGAAG
miRX132	N44ACCAAT	CGCTAGAGGTGGGTTGGAGAT
miRX13	N44CTGAAG	GCAATCGGGGGTTGACGCTT
miRX46	N44TACACT	GCGCAGGTTGAGGTCTTTAAGT
miR169b	N44CCGGCA	CGCAGCCAAGGATGACTTGC
miR397a-d	N44ACATCA	CGTCATTGAGTGCAGCGTTGA (Shen *et al.* 2014)
miRX205	N44GCCCTT	GCGTTGTAGAATTTTGGGAAGG
miR159a-c	N44TAGAGC	GCCTTTGGATTGAAGGGAGCT (Shen *et al.* 2014)
miR319a-e	N44AGGGAG	GCGTTGGACTGAAGGGAGCT (Shen *et al.* 2014)
miR827a-d	N44TATTTG	GCGCCTTAGATGACCATCAACAA (Shen *et al.* 2014)
miR845a;b	N44CATCAA	GCCGGCTCTGATACCAATTGA (Shen *et al.* 2014)
miRX136	N44TGGTGG	GACTATCTACTGCTTATGCCAC
miRX148.1	N44TTAGAG	CGTCGCGATCTTAGATCCTCT
miRX63	N44TCCGTA	GCGCATGAGTTAAGGACTTTACG
miRX95	N44GAGATC	GCGCCTTGTCGGAGTTTATGA
miRX210	N44TATGCA	GCGCTTTCTCGGAAATTCTTG
miR6032a	N44CCGAGA	GCGCTGGAGCATCAACAGAT
miRX113	N44GCCATC	GCGGGACTCGAACTCGTGAT

N44: GTCGTATCCAGTGCAGGGTCCGAGGTATTCGCACTGGATACGAC (Shen *et al.* 2014)

Universal reverse primer (5'-3'): CCAGTGCAGGGTCCGAGGT (Shen *et al.* 2014)

b) Target/Housekeeping gene expression primer

Primer name	Forward primer sequence (5'-3')	Reverse primer sequence (5'-3')
TCP4	AGGAAACGGAGGAGGAGGAG	AGATCGTCGGTGGAGATGGATTGA
XRLPK	ACAAAGTATCTGCAAACGCTAAGTC	CCAAATCCACCACCACCGAG
HACD	CACTTCCGATCTCTCCGCC	CCGTGATAGACCAGCTTATGACC
APK1	GCCTTCCAATTGCATCTCCC	GTGGCGTACATTATCTCCATCG
Actin	TCTACAACGAGCTCCGTGTTG	TGTGAGACACACCATCACCA
U6	TTGGAACGATACAGAGAAGATTAGCA(Shen *et al.* 2014)	TTGGACCATTTCTCGATTTGTG(Shen *et al.* 2014)

c) RLM-5’ RACE primers used in this study

miRNA	Target	Genespecific 3' Primer (5'-3')	Genespecific 3' Nested Primer (5'-3')
miR319a-e	TCP4	GAGAAACAGAGGAAGCAGAGGACGGTTT	CTTGACCCTGAAACCGTGCTGGCA
miRX148.1	XRLPK	GCCCATGAAAGAGGAAGCAACAC	CCTGTGGTTATCCTCGCAGCAG
miRX63	HACD	GGACCTCTGGAAAACTGTATAAGATGCC	GGCGGCGGTCTGAGCAAG
miR6032a	APK1	CTTAGCAACCTCTCCGACTCTACGAATG	CTTTCTGGTCAAGCAACCTCTGTCTATC
miR171	SCL	TCCAACGTCGAAGTCGATAATGTGG	GTTGACGAATTGAAGAAACGGCGAC
miRX27	DUF	ATAATGCCGAGAATGTAAGACACGCC	TGGAGAACTAGACCGTGATGAAGCC
miRX50	TIL	CATTATTAAAATAAAAAAACAATAGGTC	TATATCAGATTTTTTGACCCCTTGTGG
miRX137	SBT	GAGCTGATCCGCCGCCGAG	GATGTCAGATCCGAAACAGCCGCT
miRX159	PRX	GACGCATCACAACCCTGGACGAAA	CAGTCATGGAAGACAAGACGGAGGAG
miRX163	SDR	GCCAGTAGCTGGACCATTTAGAATCCC	CAACCTTTGATACCCGGAATCTCACC
	AGO1 (+ control)	CGTCTTCCAAGGTGTGACTCGCGATAC	GACCTGTCCTTTCCCAGGCCTCATT
